# Based on clinical Ki-67 expression and serum infiltrating lymphocytes related nomogram for predicting the diagnosis of glioma-grading

**DOI:** 10.3389/fonc.2022.696037

**Published:** 2022-08-25

**Authors:** Zhi Zhang, Weiguo Gu, Mingbin Hu, Guohua Zhang, Feng Yu, Jinbiao Xu, Jianxiong Deng, Linlin Xu, Jinhong Mei, Chunliang Wang, Feng Qiu

**Affiliations:** ^1^ Department of Neurosurgery, The First Affiliated Hospital of Nanchang University, Nanchang, China; ^2^ Department of Oncology, The First Affiliated Hospital of Nanchang University, Nanchang, China; ^3^ Nanchang Key Laboratory of Tumor Gene Diagnosis and Innovative Treatment Research, Nanchang, China; ^4^ Department of Oncology, Gaoxin Branch of the First Affiliated Hospital of Nanchang University, Nanchang, China; ^5^ Department of Pathology, The First Affiliated Hospital of Nanchang University, Nanchang, China; ^6^ Molecular Pathology Center, The First Affiliated Hospital of Nanchang University, Nanchang, China

**Keywords:** glioma, ki67, lymphocytes, isocitrate dehydrogenase 1, nomogram

## Abstract

**Background:**

Compelling evidence indicates that elevated peripheral serum lymphocytes are associated with a favorable prognosis in various cancers. However, the association between serum lymphocytes and glioma is contradictory. In this study, a nomogram was established to predict the diagnosis of glioma-grading through Ki-67 expression and serum lymphocytes.

**Methods:**

We performed a retrospective analysis of 239 patients diagnosed with LGG and 178 patients with HGG. Immunohistochemistry was used to determine the Ki-67 expression. Following multivariate logistic regression analysis, a nomogram was established and used to identify the most related factors associated with HGG. The consistency index (C-index), decision curve analysis (DCA), and a calibration curve were used to validate the model.

**Results:**

The number of LGG patients with more IDH1/2 mutations and 1p19q co-deletion was greater than that of HGG patients. The multivariate logistic analysis identified Ki-67 expression, serum lymphocyte count, and serum albumin (ALU) as independent risk factors associated with HGG, and these factors were included in a nomogram in the training cohort. In the validation cohort, the nomogram demonstrated good calibration and high consistency (C-index = 0.794). The Spearman correlation analysis revealed a significant association between HGG and serum lymphocyte count (r = −0.238, *P <*0.001), ALU (r = −0.232, *P <*0.001), and Ki-67 expression (r = 0.457, *P <*0.001). Furthermore, the Ki-67 expression was negatively correlated with the serum lymphocyte count (r = −0.244, *P <*0.05). LGG patients had lower Ki-67 expression and higher serum lymphocytes compared with HGG patients, and a combination of these two variables was significantly higher in HGG patients.

**Conclusion:**

The constructed nomogram is capable of predicting the diagnosis of glioma-grade. A decrease in the level of serum lymphocyte count and increased Ki-67 expression in HGG patients indicate that their immunological function is diminished and the tumor is more aggressive.

## Introduction

Gliomas continue to be one of the most prevalent and malignant primary cancer-related morbidities in the central nervous system (CNS) (1). According to the World Health Organization (WHO, 2016), low-grade glioma (LGG) is classified as grade I–II, while high-grade glioma (HGG) is classified as grade III-IV (2). Although glioblastomas (GBMs, grade IV) are treated with maximally safe surgical resection and combined radio-chemotherapy, the median overall survival (OS) is 15 months and the 5-year survival rate is less than 5% due to the complexity of tumors, widespread invasiveness, and heterogeneity (3, 4). Furthermore, several studies have demonstrated that approximately 20%–25% of secondary glioblastomas are derived from previously lower grade (WHO grades II or III) gliomas (5, 6). Therefore, numerous studies indicate that the basic clinical–pathological features of serum laboratory indices or immunohistochemical (IHC) staining are significant variables in identifying secondary glioblastomas (7–10).

Histological, molecular features, and anatomical sites play an important role in the classification and diagnosis of glioma by the fifth edition of the WHO Classification of Tumors of the CNS (WHO CNS5) (11). The isocitrate dehydrogenase (IDH) gene encodes an enzyme that is involved in the control of cellular metabolism, epigenetic regulation, redox states, and DNA repair (12). The IDH mutation is critical for diagnosis, treatment efficacy evaluation, survival prediction, and reduced invasiveness of biomarkers associated with glioma, and is widely deemed the most significant genetic alteration (13, 14). It is associated with better outcomes in IDH1-mutant patients than in IDH1 wild-type patients (15, 16). Therefore, according to the new classification, all IDH-mutant diffuse astrocytic tumors are considered a single type (astrocytoma, IDH-mutant) and are graded as 2, 3, or 4. The restriction of the diagnosis of glioblastoma to IDH wild-type tumors means that IDH-mutant gliomas are not GBMs anymore (11, 17). This is perhaps one of the most important changes to the older version of the 2016 WHO classification. Theresia et al. found an association between the Ki-67 labeling index and histopathological grading of glioma. LGG patients had a significantly lower Ki-67 labeling index than HGG patients, and a cut-off of 10% was used to differentiate LGG from HGG (18, 19). The relationship between Ki-67 expression and IDH1 mutation status demonstrated by Zeng et al. (20) showed that Ki-67 expression along with IDH1/2 can significantly differentiate prognosis in glioma, with low Ki-67 expression associated with increased IDH1/2 mutation and IDH1/2 mutant patients with low and moderate Ki-67 expression having the best prognosis. However, it is not known whether Ki-67 expression along with IDH1 can differentiate glioma-grading.

Tumor immune infiltration of the microenvironment with inflammatory factors is critical for the occurrence, development, and prognosis of glioma. Tumor locations, in particular, release immune infiltrates and inflammatory factors into the peripheral blood, triggering an inflammatory immune response that might provide prognostic information. Accumulating evidence suggests that elevated peripheral blood lymphocytes are associated with a favorable prognosis, particularly in lung, breast, and colorectal cancers (21–23). Additionally, macrophages originating from bone marrow mononuclear cells generate an inflammatory immune response, including the release of pro-inflammatory factors such as TNF-α, chronic factors IL-6 and IL-1, which migrate to the glioma site across the blood–brain barrier (24, 25). Numerous studies indicate that T-lymphocyte subsets may affect the prognosis of breast, melanoma, pancreatic, and colorectal cancers (21–23, 26). Kmiecik et al. (27) found a correlation between tumor-infiltrating lymphocytes and prolonged survival in GBS patients. However, Zhao et al. (28) found that local tumor-infiltrating lymphocytes were a poor prognostic marker in GBS. Therefore, the correlation between peripheral serum lymphocyte or lymphocyte infiltration at the tumor site and glioma grade or prognosis is contradictory, and only a few studies have been published.

Finally, the relationship between the immunohistochemical index of IDH1, Ki-67 expression, and the peripheral serum lymphocyte count in glioma grading is unknown. Therefore, we conducted a retrospective study to determine the prognostic usefulness of preoperative peripheral serum lymphocyte count, postoperative immunohistochemical index of IDH1 mutation status, and Ki-67 expression for glioma grading. Because nomograms are widely used to predict the risk of cancer, we established a nomogram that uses Ki-67 expression and serum lymphocyte count to predict the differences between LGG and HGG. This nomogram will aid clinical doctors in predicting the glioma-grading and identifying potential risk factors for HGG patients and allow for early treatment intervention.

## Materials and methods

### Patients and data collection

Between January 2012 and December 2020, we conducted a retrospective review of glioma patients. This study was approved by the Ethics Committee of the First Affiliated Hospital of Nanchang University. The WHO classifies glioma grades I–II as LGG and grades III–IV as HGG. Data of patients included their age, sex, immunohistochemistry index, serum clinical laboratory indicators, and glioma grade. The inclusion criteria were as follows: 1) all patients were admitted for surgery and histologically-confirmed glioma postoperatively; and 2) patients with complete information and Ki-67 testing. The exclusion criteria were as follows: 1) preoperative chemoradiotherapy or incomplete information; 2) along with other malignant tumors; and 3) patients with previous blood system diseases and infection or antibiotic use.

### Establishment and validation of the nomogram

**Figure 1:**
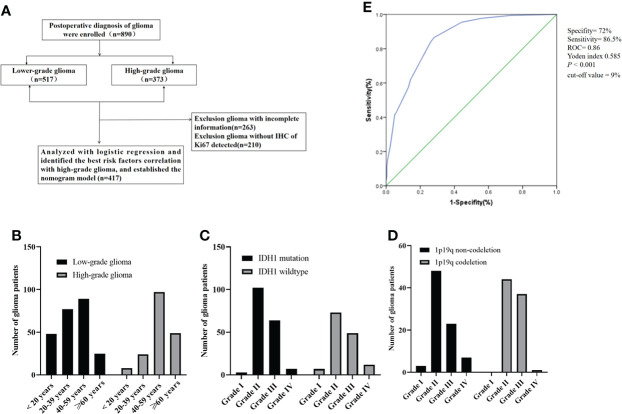
**(A)** Consort diagram, enrollment, and outcome; **(B)** the age distribution of patients with glioma; **(C)** IDH1 mutation status of patients with glioma; **(D)** the number of glioma patients with 1p19q codeletion status; **(E)** the Ki-67 curve of cut-off value (according to the LGG and HGG). ROC, Receiver operating characteristic; IDH1, Isocitrate dehydrogenase 1.

The R package “rms” was used to randomly divide the patients into two groups in a ratio of 2:1 training cohort (n = 292) and validation cohort (n = 125) (**Figure 1A**). A nomogram was established in the training cohort using multivariate logistic regression analysis, which revealed the most important predictive risk factors associated with HGG. The consistency index (C-index) ranged between 0.5 (no discrimination) and 1 (perfect discrimination), and a high C-index indicated a good prediction model. The calibration curve was used to determine the prediction compliance, while the decision curve analysis (DCA) was used to assess the clinical utility and threshold probability of the model.

### Statistical analysis

The continuous variables were represented as mean ± standard deviation, and if the variables followed a normal distribution, the comparison between the two groups was carried out using the Student’s t-test. To perform univariate analysis, continuous variables were converted to categorical variables. Univariate and multivariate logistic regression analyses were used to determine the independent risk factors as well as the odds ratio (OR) and 95% confidence interval (CI). The Spearman correlation coefficient was used to determine the correlation between the independent risk factors and HGG. IBM SPSS 22.0 software (SPSS Inc., Chicago, IL, USA) and GraphPad Prism version 8.0 software (Inc., La Jolla, CA, USA) were used to analyze the data. R statistical software version 4.0.0 (http://www.R-project.org/) was used to construct the nomogram model, calibration curve, and DCA. The optimal cut-off values for Ki-67 expression were determined by plotting the receiver operating characteristic (ROC) curves for glioma grading. The normal serum lactate dehydrogenase (LDH) levels were determined using appropriate assay kits (29, 30). *P <*0.05 was considered statistically significant.

## Results

### Correlation between glioma and clinicopathological characteristics

A total of 417 (239 with LGG and 178 with HGG) patients were postoperatively diagnosed with glioma and did not receive any treatment preoperatively. The clinicopathological features of glioma are summarized in **Table 1**. Serum white blood cells (WBCs), lymphocytes, neutrophils, neutrophil-to-lymphocytes ratio (NLR), platelets (PLTs), platelet-to-lymphocytes ratio (PLR), albumin (ALU), and LDH were used as hematological markers. There were 13 cases of grade I glioma, 226 cases of grade II glioma, 157 cases of grade III glioma, and 21 cases of grade IV glioma. The median age of the patients was 45 years (a range of 3–79). The relationship between age distribution and glioma grading revealed that the morbidity in LGG patients was mostly in the 20–59 year age range, while in HGG patients it was primarily in the more than 40 year age range (**Figure 1B**). A total of 141 IDH1 mutant patients were identified, 176 patients were identified as IDH1 wild-type, and 100 patients were identified as unknown. A total of 82 patients had co-deletion of chromosome 1 and the long arm of chromosome 19 (1p19q), 81 patients had non-codeletion, and 254 were unknown. There were more IDH1 mutation patients in grades II and III than in grades I and IV, with grade II having the highest number of patients with IDH1 mutations (**Figure 1C**). The number of grade II and III patients with 1p19q codeletion was more than those in grades I and IV, and there were no grade I patients with 1p19q codeletion (**Figure 1D**). **Figure 1E** shows the cut-off point for the Ki-67 ROC curve calculated using glioma grading (by LGG and HGG). Because the cut-off value of 9% had the highest sensitivity and specificity (sensitivity was 86.5%, specificity was 72%, Yoden index 0.585, ROC = 0.86, *P <*0.001), we divided the Ki-67 into low and high groups using a cut-off value of 10%.

**Table 1:** Demographic and clinical–pathological characteristics of the training cohort and validation cohort.

Characteristic	Groups	All cohort (n = 417, %)	Training cohort (n = 292, %)	Validation cohort (n = 125, %)
Gender	Male	230 (55.2)	160 (54.8)	70 (56.0)
	Female	187 (44.8)	132 (45.2)	55 (44.0)
Age (years)	<60	343 (82.3)	235 (80.5)	108 (86.4)
	≥60	74 (17.7)	57 (19.5)	17 (13.6)
Tumor diameter	≤2 cm	68 (16.3)	46 (15.8)	22 (17.6)
	>2 cm	349 (83.7)	246 (84.2)	103 (82.4)
Glioma grades	WHO I	13 (3.1)	6 (2.1)	7 (5.6)
	WHO II	226 (54.2)	161 (55.1)	65 (52)
	WHO III	157 (37.7)	112 (38.4)	45 (36)
	WHO IV	21 (5)	13 (4.4)	8 (6.4)
IDH1 mutation	Wilds	141 (33.8)	96 (32.9)	45 (36)
	Mutations	176 (42.2)	118 (40.4)	58 (46.4)
	Unknown	100 (24.0)	78 (26.7)	22 (17.6)
1p/19q codeletion	Negative	81 (19.4)	58 (19.9)	23 (18.4)
	Positive	82 (19.7)	56 (19.2)	26 (20.8)
	Unknown	254 (60.9)	178 (60.9)	76 (60.8)
Ki-67^a^	≤10%	273 (65.5)	202 (69.2)	71 (56.8)
	>10%	144 (34.5)	90 (30.8)	54 (43.2)
ATRX	Negative	24 (5.8)	15 (5.1)	9 (7.2)
	Positive	87 (20.9)	63 (21.6)	24 (19.2)
	Unknown	306 (73.3)	214 (73.3)	92 (73.6)
CD56	Negative	5 (1.2)	5 (1.7)	0 (0)
	Positive	272 (65.2)	190 (65.1)	82 (65.6)
	Unknown	140 (33.6)	97 (33.2)	43 (34.4)
P53	Negative	96 (23)	68 (23.3)	28 (22.4)
	Positive	141 (33.8)	93 (31.8)	48 (38.4)
	Unknown	180 (43.2)	131 (44.9)	49 (39.2)
EMA	Negative	247 (59.2)	175 (59.9)	72 (57.6)
	Positive	60 (14.4)	38 (13)	22 (17.6)
	Unknown	110 (26.4)	79 (27.1)	31 (24.8)
GFAP	Negative	15 (3.6)	11 (3.8)	4 (3.2)
	Positive	279 (66.9)	195 (66.8)	84 (67.2)
	Unknown	123 (29.5)	86 (29.4)	37 (29.6)
WBC (10^9^/L)^b^	≤7.07	255 (61.2)	178 (61.0)	77 (61.6)
	>7.07	162 (38.8)	114 (39.0)	48 (38.4)
RBC (10^12^/L)^b^	≤4.54	212 (50.8)	153 (52.4)	59 (47.2)
	>4.54	205 (49.2)	139 (47.6)	66 (52.8)
HB (g/L)^b^	≤134	202 (48.4)	142 (48.6)	60 (48.0)
	>134	215 (51.6)	150 (51.4)	65 (52.0)
PLT (10^9^/L)^b^	≤223	216 (51.8)	155 (53.1)	61 (48.8)
	>223	201 (48.2)	137 (46.9)	64 (51.2)
Lymphocyte (10^9^/L)^b^	≤1.7	216 (51.8)	149 (51.0)	67 (53.6)
	>1.7	201 (48.2)	143 (49.0)	58 (46.4)
Neutrophils (10^9^/L)^b^	≤4.78	270 (64.7)	193 (66.1)	77 (61.6)
	>4.78	147 (35.3)	99 (33.9)	48 (38.4)
NLR^b^	≤3.6	323 (77.5)	230 (78.8)	93 (74.4)
	>3.6	94 (22.5)	62 (21.2)	32 (25.6)
PLR^b^	≤152.7	259 (62.1)	190 (65.1)	69 (55.2)
	>152.7	158 (37.9)	102 (34.9)	56 (44.8)
ALU(g/L)	≤42.7	214 (51.3)	150 (51.4)	64 (51.2)
	>42.7	203 (48.7)	142 (48.6)	61 (48.8)
LDH (U/L) ^c^	≤250	318 (76.3)	220 (75.3)	98 (78.4)
	>250	99 (23.7)	72 (24.7)	27 (21.6)

IDH1, isocitrate dehydrogenase-1; 1p/19q co-deletion, chromosome 1 and the long arm of chromosome 19; ATRX, alpha-thalassemia/mental retardation syndrome X-linked; Ki-67, nuclear proliferation antigen 67; GFAP, glial fibrillary acidic protein; EMA, epithelial membrane antigen; PLR, platelet-to-lymphocyte ratio; NLR, neutrophil-to-lymphocyte ratio; ALU, albumin; LDH, lactate dehydrogenase; WHO, World Health Organization. ^a^The cut-off points was used by ROC curve (according to LGG and HGG); ^b^The cut-off points was used mean value; ^c^The cut-off points was used relevant assay kits, and all those factors divided into high and low groups for statistical analysis.

### Univariate and multivariate logistic analyses in the training cohort

All glioma patients were randomly assigned to the training cohort (n = 292) or the validation cohort (n = 125) using the R package “rms.” There were 167 patients with LGG and 125 with HGG in the training cohort. Univariate logistic analysis revealed a significant correlation between age, Ki-67 expression, NLR, serum lymphocyte count, serum ALU, and glioma-grading (*P <*0.05) (**Table 2**). All significant factors in the univariate analysis were included in the multivariate logistic regression analysis. The result showed that Ki-67 >10%, serum lymphocytes count ≤1.7 (×10^9^/L), and serum ALU ≤42.7 g/L were all independent risk factors associated with HGG (*P <*0.05) **(** **Table 3**).

**Table 2:** Univariate logistic proportional hazards regression analysis in the training cohort.

Characteristic		Glioma		OR (95% CI)	*P-value*
		Low grades	High grades		
Gender	Male	89	71	Ref	
	Female	78	54	0.868 (0.544–1.384)	0.551
Age (years)	<60	148	87	Ref	
	≥60	19	38	3.402 (1.847–6.268)	<0.001
Tumor diameter	≤2 cm	29	17	Ref	
	>2 cm	138	108	1.335 (0.697–2.556)	0.383
IDH1 mutation	Negative	58	38	Ref	
	Positive	68	50	1.122 (0.649–1.942)	0.68
1p/19q codeletion	Negative	37	21	Ref	
	Positive	30	26	1.527 (0.721–3.233)	0.269
Ki-67	≤10%	146	56	Ref	
	>10%	21	69	8.566 (4.808–15.262)	<0.001
ATR-X	Negative	9	6	Ref	0.841
	Positive	36	27	1.125 (0.357–3.543)	
CD56	Negative	4	1	Ref	
	Positive	104	86	3.308 (0.363–30.147)	0.289
P53	Negative	44	24	Ref	
	Positive	47	46	1.794 (0.944–3.411)	0.074
EMA	Negative	100	75	Ref	
	Positive	21	17	1.079 (0.533–2.187)	0.832
GFAP	Negative	5	6	Ref	
	Positive	116	79	0.568 (0.167–1.924)	0.363
WBC (10^9^/L)^a^	≤7.07	102	76	Ref	
	>7.07	65	49	1.012 (0.629–1.627)	0.962
RBC (10^12^/L)^a^	≤4.54	80	73	Ref	
	>4.54	87	52	0.655 (0.41–1.046)	0.072
HB (g/L)^a^	≤134	76	66	Ref	
	>134	91	59	0.747 (0.469–1.188)	0.218
PLT (10^9^/L)^a^	≤223	81	74	Ref	
	>223	86	51	0.649 (0.406–1.037)	0.071
Lymphocyte (10^9^/L)^a^	≤1.7	68	81	Ref	
	>1.7	99	44	0.373 (0.231–0.603)	<0.001
Neutrophils (10^9^/L)^a^	≤4.78	115	78		
	>4.78	52	47	1.333 (0.818–2.171)	0.249
NLR^a^	≤3.6	142	88	Ref	
	>3.6	25	37	2.388 (1.347–4.235)	0.003
PLR^a^	≤152.7	115	75	Ref	
	>152.7	52	50	1.474 (0.908–2.395)	0.117
ALU (g/L)	≤42.7	69	81	Ref	
	>42.7	98	44	0.382 (0.237–0.618)	<0.001
LDH (U/L) ^b^	≤250	126	94	Ref	
	>250	41	31	1.013 (0.592–1.735)	0.961

IDH1, isocitrate dehydrogenase-1; 1p/19q co-deletion, chromosome 1 and the long arm of chromosome 19; ATRX, alpha-thalassemia/mental retardation syndrome X-linked; Ki67, nuclear proliferation antigen 67; GFAP, glial fibrillary acidic protein; EMA, epithelial membrane antigen; PLR, platelet-to-lymphocyte ratio; NLR, neutrophil-to-lymphocyte ratio; ALU, albumin; LDH, lactate dehydrogenase.

**Table 3:** Multivariate logistic proportional hazards regression analysis in the training cohort.

Characteristic	Groups	OR (95% CI)	*P*-value
Ki-67	≤10%	Ref	
	>10%	8.758 (4.754–16.136)	<0.001
Lymphocyte (10^9^/L)	≤1.7	Ref	0.003
	>1.7	0.436 (0.252–0.755)	
ALU (g/L)	≤42.7	Ref	0.001
	>42.7	0.378 (0.217–0.659)	

Ki67, nuclear proliferation antigen 67; ALU, albumin.

### Correlation analysis between the independent risk factors and HGG in all cohorts

**Figure 2:**
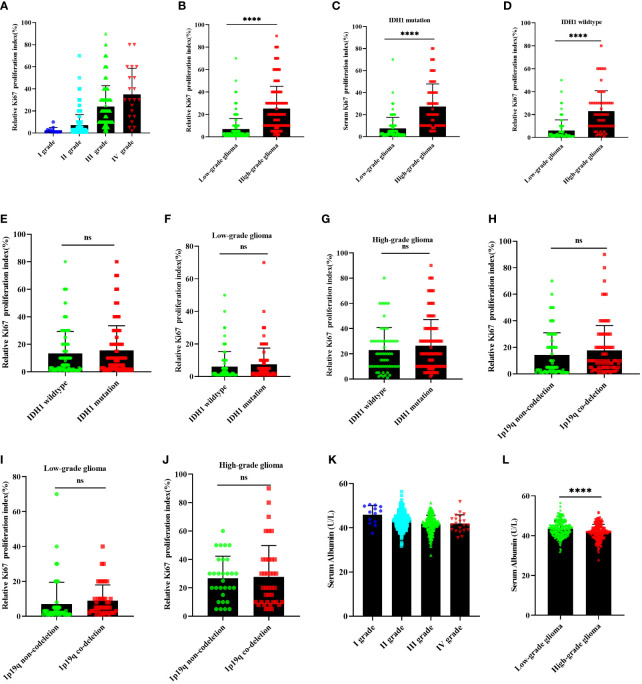
The relationship between Ki-67 expression and glioma, IDH1 mutation status, 1p19q co-deletion status. **(A, B)** the Ki-67 expression in glioma; **(E–G)** Ki-67 expression correlation with IDH1 mutation status in all glioma, LGG, and HGG; **(H–J)** Ki-67 expression correlation with 1p19q co-deletion status in all glioma, LGG, and HGG; **(C, D)**, Ki-67 expression correlation with glioma in IDH1 mutation group and IDH1 wild type group; **(K, L)** serum ALU correlation with glioma. LGG, lower-grade glioma; HGG, high-grade glioma; ALU, albumin. ^ns^ *P* ^>^0.05, ****P <0.0001, mean ± standard deviation, t-test.

The Spearman correlation analyses were used to determine the correlation between the independent risk factors and HGG in all cohorts. The serum lymphocyte count (r = −0.238, *P <*0.001), ALU (r = −0.232, *P <*0.001), and Ki-67 expression (r = 0.457, *P <*0.001) were all shown to be significantly associated with glioma-grading **(** **Table 4**). Ki-67 expression increased gradually from grades I to IV and was significantly higher in HGG patients than in LGG patients (*P <*0.05), regardless of glioma type, IDH1 mutation, or wild type (**Figures 2A–D** **)**. We performed subgroup analysis to determine the correlation between IDH1, 1p19q codeletion, and Ki-67 expression. The result indicated that Ki-67 expression was not associated with IDH1 mutation or 1p19q codeletion **Figures 2C–H**). Additionally, serum ALU levels significantly decreased from grades I to IV, and the LGG group had better nutritional status than those in the HGG group (**Figures 2K–L**).

**Table 4:** Spearman correlation analysis independent risk factors with HGG in all cohorts.

Spearman		Ki-67	Lymphocyte	ALU
Glioma	r	0.457	−0.238	−0.232
	*P*	<0.001	<0.001	<0.001

**Figure 3:**
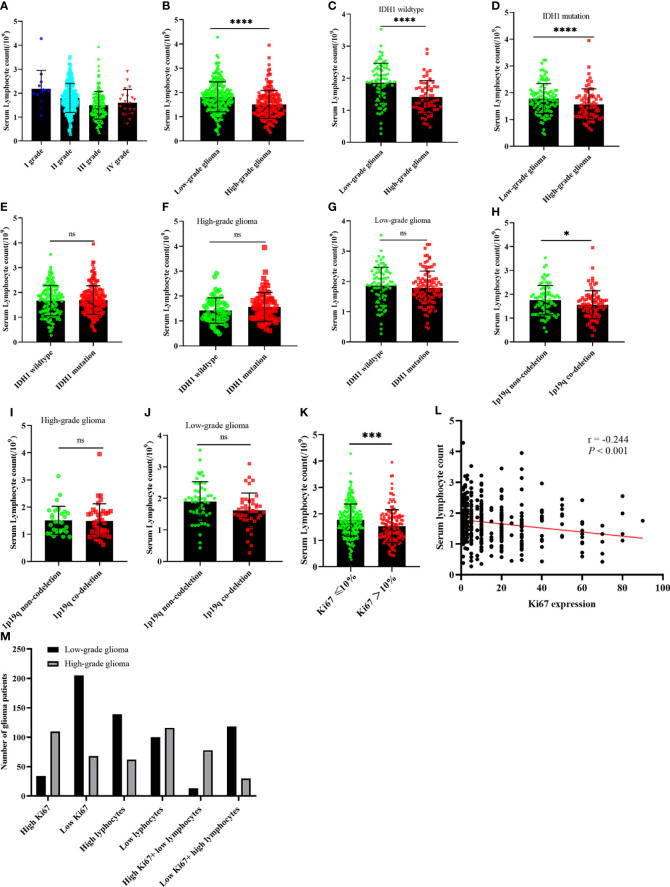
The relationship between serum lymphocyte count and glioma, IDH1 mutation status, 1p19q co-deletion status, and serum ALU correlation with glioma. **(A, B)** serum lymphocyte count in glioma; **(C, D)** serum lymphocyte count correlation with glioma in IDH1 mutation group and IDH1 wild-type group; **(E–G)** serum lymphocyte count correlation with IDH1 mutation status in all glioma, HGG, and LGG; **(H–J)** serum lymphocyte count correlation with 1p19q co-deletion status in all glioma, HGG, and LGG; **(K)** serum lymphocyte count correlation with Ki-67 expression in all glioma; **(L)** linear correlation between serum lymphocyte count and Ki-67 expression; **(M)** the number of patients with Ki-67 expression and serum lymphocytes count. LGG, lower-grade glioma; HGG, High-grade glioma; ALU, albumin. ^ns^ *P* ^>^0.05, *P <0.05, ***P <0.001, ****P <0.0001, mean ± standard deviation, t-test.

The serum inflammatory index plays an important role in the differentiation and proliferation of tumor cells. Therefore, we examined the relationship between serum lymphocyte count and glioma grade. The serum lymphocyte count significantly decreased from grades I to IV (**Figure 3A**). The serum lymphocyte count in LGG patients was significantly higher than in HGG patients, regardless of glioma type, IDH1 mutation, or wild-type status (*P*<0.05) (**Figures 3B–D**). Additionally, we performed a subgroup analysis of serum lymphocytes according to their IDH mutation status or 1p19q co-deletion status. Regardless of LGG or HGG status, the serum lymphocyte count in 1p19q codeletion groups, as well as in IDH1 mutation or wild-type groups, was not correlated with them (*P >*0.05) (**Figures 3E–J**). The serum lymphocytes in high Ki-67 expression groups were significantly lower than serum lymphocytes in low Ki-67 expression groups in all glioma patients (**Figure 3K**); the two groups exhibited a negative correlation (**Figure 3L**). We then analyzed the diagnostic value of Ki-67 expression and serum lymphocyte count in glioma. The HGG patients were mostly classified as having a high Ki-67 expression and a low lymphocyte count. The Ki-67 index, along with serum lymphocytes, may be used to distinguish LGG from HGG. This approach may be critical in the diagnosis of glioma patients (**Figure 3M**).

### Construction and validation of the nomogram

**Figure 4:**
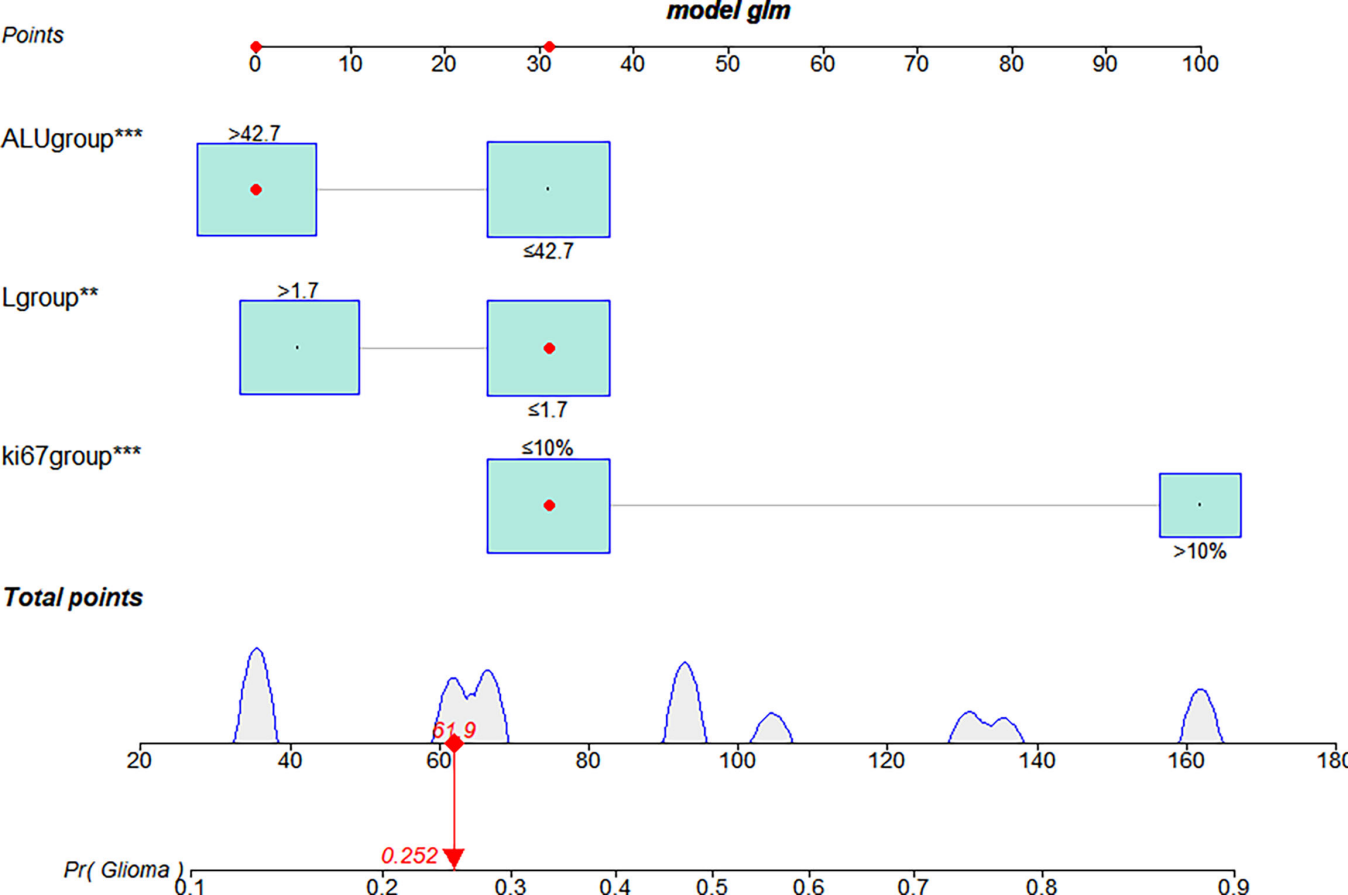
The nomogram used to predict the glioma grading in the training cohort. Three independent risk factors were incorporated into the nomogram model, and the data for those variables are shown on the interactive nomogram. Each predictive variable had a value ranging from 0 to 100, and the overall score was calculated by summing those variables. The red dot on the scale represents the corresponding score of the variable. LGG, lower-grade glioma; HGG, High-grade glioma; ALU, albumin. **P <0.01, ***P <0.001.

The multivariate logistic analysis identified Ki-67 expression, serum lymphocyte count, and serum albumin (ALU) as independent risk factors associated with HGG in the training cohort, and these variables were included in a nomogram. The weight of each variable was assigned a value between 0 and 100, and the HGG possibility was calculated as a sum of the corresponding scores shown on the coordinates (**Figure 4**).

**Figure 5:**
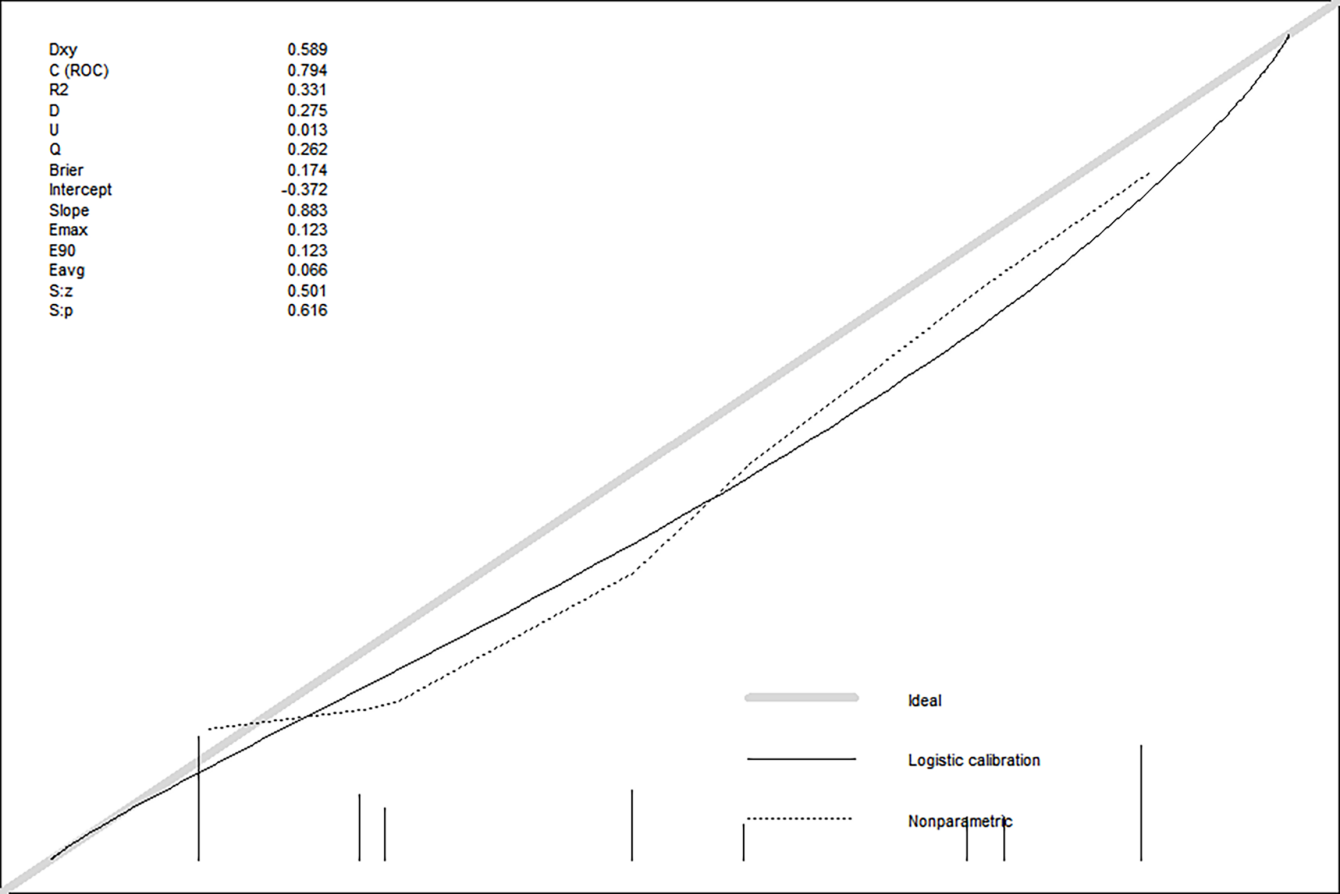
The discrimination and calibration curves of prediction model.

**Figure 6:**
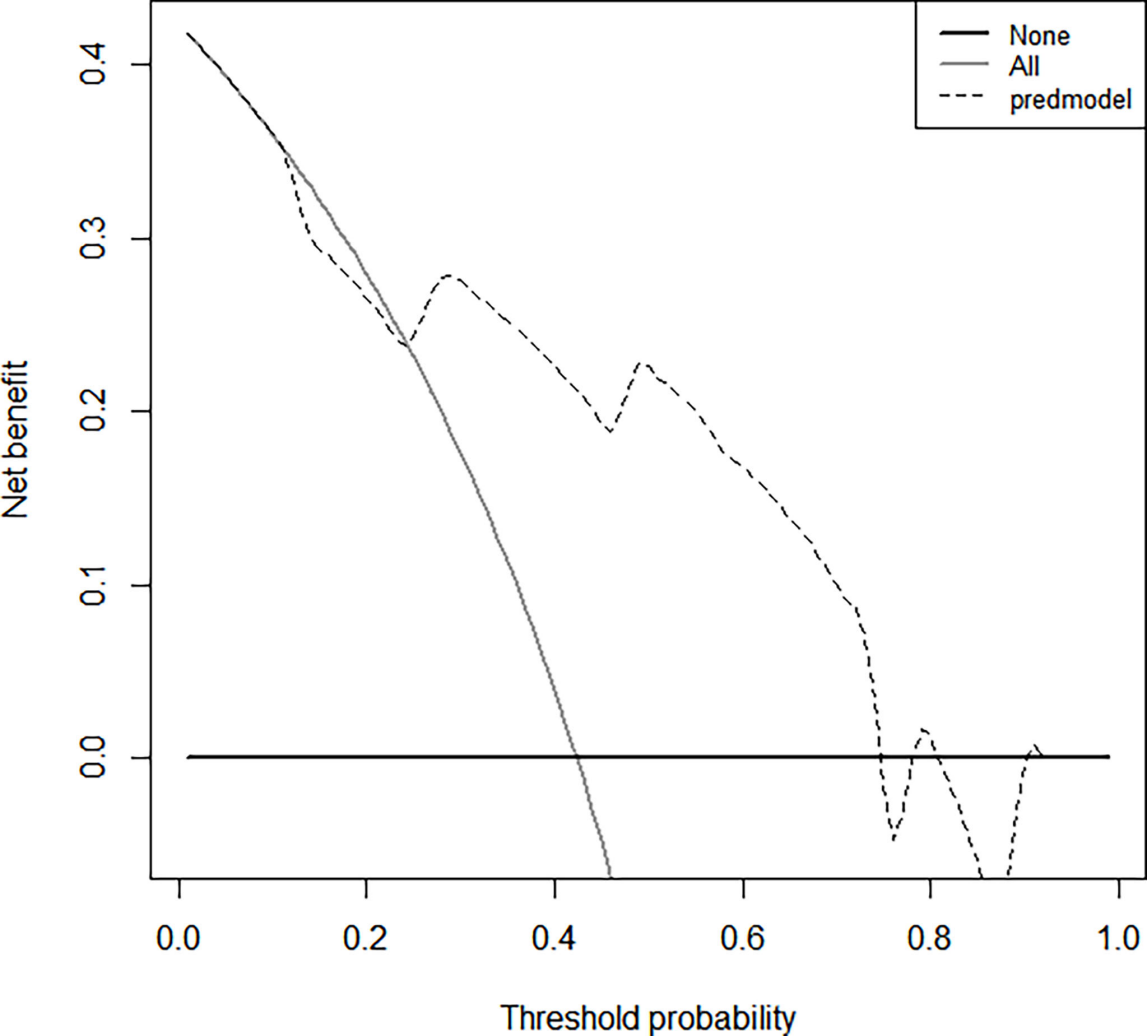
The clinical values of this nomogram model for decision curve analyses (DCA). The Y-axis represents the net benefit. The dotted line represents the clinicopathologic nomogram. The gray line represents the hypothesis that all patients are involved in HGG. The black solid line represents the hypothesis that no patients are involved in HGG. The X-axis represents the HGG possibility (31).

The bootstrap c-index of the nomogram was 0.794 (0.71–0.90), indicating that the nomogram model established had a high degree of accuracy in distinguishing LGG from HGG patients. Additionally, the calibration curve indicated that the regression fitting curve was very close to the standard curve and that there was no statistically significant difference between the two curves (*P* = 0.616), indicating that the model had a high degree of calibration and was very close to the actual outcome (**Figure 5**). Additionally, the DCA demonstrated that the clinical value of the model presented more net benefits at 30%–73% and 78%–82% threshold probabilities, indicating that the postoperative LGG patients with high-risk factors who received treatment had a greater net benefit than either the treat all patients or treat none patients (**Figure 6**).

## Discussion

Numerous studies have demonstrated that approximately 20%–25% of LGG can develop into HGG and eventually lead to death, and the 5-year survival rate of HGG is less than 5% (4, 32). Due to the limitations of imaging technology, we were unable to detect micrometastasis sites in local tumor lesions even when sophisticated magnetic resonance imaging (MRI) was used to examine the postoperative LGG patients, resulting in the patients missing out on their best opportunity for therapy. IHC and serum Systemic Inflammatory Reaction (SIR) have been shown in several studies to play an essential role in glioma grading and prognosis (7–10). Therefore, MRI combined with immunohistochemistry and blood inflammatory biomarkers will be a highly effective method for predicting HGG in future studies.

In this study, we collected the postoperative IHC and preoperative serum inflammatory-related indicators and establish a correlation with HGG. We found that the Ki-67 expression gradually increased, but the peripheral blood lymphocyte count decreased with the grading of gliomas. Spearman correlation analysis showed that Ki-67 expression had a negative linear correlation with serum lymphocytes, thus, high Ki-67 expression was associated with a lower serum lymphocyte count. Furthermore, the nomogram model was established using Ki-67 and serum lymphocytes, and it was found to be highly accurate in predicting the HGG. This is the first study to incorporate Ki-67 expression, serum lymphocytes, and clinicopathological factors in predicting the glioma-grading, which may help clinical doctors in identifying potential risk factors for HGG patients.

The SIR tumor immune infiltration microenvironment releases immune cytokines and inflammatory factors into the peripheral blood, activating the inflammatory immune response, which is critical for regulating proliferation, invasion, distant metastasis, and prognosis in lung cancer, breast cancer, colon cancer, and glioma (21–25). This study discovered a correlation between the preoperative neutrophil-lymphocyte ratio (NLR) and glioma grade, and that an elevated NLR was an independent predictor of poor outcome in glioblastoma patients (33). Marinari et al. (34) found that peripheral immune signatures associated with increased inflammation, immune infiltration, and activation were associated with poor survival in HGG patients, and that lymphocyte infiltration at the tumor site was also associated with poor survival, implying that immune responses may play a pro-tumorigenic role in glioma. Kmiecik et al. (27) described the mechanisms of immunological escape in GBM, demonstrating that increased CD3(+) tumor-infiltrating T-lymphocyte cells were associated with prolonged survival in GBM patients and were also correlated with integrated immunosuppressive mechanisms in the tumor microenvironment and at the systemic level. Therefore, the association between tumor location and peripheral blood infiltrating lymphocytes in glioma is controversial. In this study, we found that the peripheral blood infiltrating lymphocyte count in LGG was higher than in HGG, and that grade I had the highest serum lymphocyte count. Additionally, a low serum lymphocyte count is an independent risk factor for HGG. Numerous studies have also demonstrated that IDH mutations play an important role in diagnosing, evaluating medication effectiveness, predicting survival, and reducing the invasiveness of biomarkers associated with glioma, and are widely deemed the most significant genetic alteration (13–16). Then, we further performed a correlation analysis between serum lymphocyte count and IDH1 mutation or 1p19q codeletion. In HGG or LGG patients, serum lymphocyte count showed no correlation with IDH1 mutation or 1p19q codeletion.

Additionally, we examined the IHC expression of Ki-67 and serum lymphocytes in glioma. The Ki-67 expression was gradually elevated with glioma grade, and the GBM had the highest Ki-67 expression, however, there was no association between the IDH1 mutation or 1p19q codeletion status and Ki-67 expression. Spearman correlation analysis revealed a negative linear association between Ki-67 expression and serum lymphocytes, with a higher Ki-67 expression corresponding to a lower serum lymphocyte count. Then, we examined the diagnostic utility of Ki-67 expression in glioma by combining with the serum lymphocyte count. We discovered that LGG patients have lower Ki-67 expression and a higher blood lymphocyte count than HGG patients and that a combination of these two factors may significantly distinguish LGG from HGG, perhaps playing a role in diagnosing HGG patients. Taken together, a decrease in serum lymphocytes and increased expression of Ki-67 in HGG patients indicates that the tumor immune capacity of patients is diminished and tumors are more aggressive, which may contribute to the overall survival of LGG patients being longer than HGG patients. Furthermore, there was no association between Ki-67 expression and IDH1 mutation or 1p19q codeletion. As several studies have demonstrated that overexpression of Ki-67 increases tumor proliferation, invasion, and metastasis, it is also a critical reference index for the diagnosis and prognosis of breast cancer, lung cancer, and prostate cancer (35–39). Theresia et al. (18) demonstrated an association between the Ki-67 labeling index and the histopathological grade of glioma, with LGG having a significantly lower grade than HGG. Ki-67 expression may be utilized to quantify lymphocyte proliferation (40). Li et al. (41) demonstrated a negative correlation between pre-and postoperative expression and alterations of peripheral blood lymphocytes and their CD25 and Ki-67 expression in renal cell carcinoma cells using flow cytometry and immunohistochemistry. However, no investigation has been reported to determine the correlation between Ki-67 expression and serum lymphocytes.

Nomograms are widely used for predicting the risk of cancer, and using basic hematological and clinicopathological data to identify risk factors for survival prediction is also a useful and valuable tool in tumors (42, 43). Wu et al. (44) established and validated a novel nomogram for the preoperative diagnosis of GBM using feasible baseline characteristics and preoperative tests. The nomogram demonstrated excellent calibration and a significant clinical advantage in predicting GBM. Another study suggested that a nomogram based on inflammatory biomarkers can accurately predict overall survival rates in patients with glioma, with a high NLR rate associated with a poor prognosis (45). However, no relevant studies have been reported using the Ki-67 expression and serum lymphocytes of the nomogram to predict the risk of glioma grade. In this study, univariate and multivariate logistic regression analysis revealed that Ki-67 expression and serum lymphocytes were independent risk factors for HGG, and the established nomogram may be used to accurately predict HGG. This is the first study to assess the predictive value of Ki-67 expression and serum lymphocytes in patients with glioma.

There are some limitations to our study. Firstly, this study only examined the correlation between peripheral blood lymphocyte count and glioma grade, but not the survival of patients following surgery or adjuvant chemoradiotherapy. Secondly, we only examined the relationship between serum lymphocytes and IDH1 mutation or 1p19q codeletion status. However, the overall survival was unclear based on both mutation status and analysis of serum lymphocytes. Thirdly, we analyzed and verified the correlation between peripheral lymphocytes and HGG retrospectively, but the expression of O6-methylguanine-DNA methyltransferase (MGMT), CD3, CD4, or CD8, etc. in peripheral blood T-lymphocytes and which inflammatory factors were released and passed through the blood–brain barrier to influence tumors were unknown. Additionally, we classified glioma-grade and pathological classification according to WHO 2016 guidelines; nevertheless, the most recent pathological classification of glioma has undergone significant changes. Finally, because this was a single-center, retrospective study with a limited sample size and a small number of grade I or IV glioma patients, the results may have been subject to selection bias. Therefore, a larger sample size, multi-centered clinical study of serum lymphocytes with glioma grade should be considered in the future.

In conclusion, the established nomogram may be used to predict HGG, and the HGG patients with greater serum lymphocyte counts and lower Ki-67 expression. The reduction in serum lymphocytes and increased expression of Ki-67 in HGG patients indicate that their immunological function is compromised and their tumors are more aggressive. Moreover, IHC of Ki-67 expression along with serum lymphocytes may accurately detect HGG. Therefore, we will further conduct long-term follow-up of patients and predict the risk of whose LGG will transform into HGG in the future. This may be useful in assisting clinical doctors in predicting secondary glioma in patients at high risk of postoperative LGG and allowing for early treatment intervention.

## Data availability statement

The raw data supporting the conclusions of this article will be made available by the authors, without undue reservation.

## Ethics statement 

The studies involving human participants were reviewed and approved by the First Affiliated Hospital of Nanchang University, Nanchang, China. The ethics committee waived the requirement of written informed consent for participation. All the patients’ data are keep confidential.

## Author contributions

WG and ZZ were involved in collected the data and patient follow-up. WG and MH were responsible for the conception and design of the study, assisted with the statistical analysis and wrote the manuscript. CW, FQ, and JM contributed in the correction of the manuscript. LX, FY, JX, JD, and GZ helped correcting and revising the manuscript. All authors approved the manuscript prior to submission.

## Funding

This work was supported by the National Natural Science Foundation of China (81960495 to FQ, 81760448 to CW) and the Nanchang Key Laboratory of Tumor Gene Diagnosis and Innovative Treatment Research (2021-NCZDSY-009). The funding sources had no role in the data collection, analysis or interpretation.

## Acknowledgments

We appreciate the effort of the physicians for enrolling patients and thank all the patients involved for allowing us to analyze their clinical data.

## Conflict of interest

The authors declare that the research was conducted in the absence of any commercial or financial relationships that could be construed as a potential conflict of interest.

## Publisher’s note

All claims expressed in this article are solely those of the authors and do not necessarily represent those of their affiliated organizations, or those of the publisher, the editors and the reviewers. Any product that may be evaluated in this article, or claim that may be made by its manufacturer, is not guaranteed or endorsed by the publisher.
